# The Clinical Role of the Adenoid Microbiome in the Development of Adenoid Hypertrophy and Otitis Media with Effusion

**DOI:** 10.3390/jcm15062187

**Published:** 2026-03-13

**Authors:** Oļegs Sokolovs-Karijs, Monta Brīvība, Dita Gudrā, Rihards Saksis, Annija Alise Zodāne, Maija Rozenberga, Laura Bunka, Anna Frigotto, Jana Osīte, Aigars Reinis, Gunta Segliņa, Angelika Krūmiņa

**Affiliations:** 1 Department of Otolaryngology, Riga Stradiņš University, 16 Dzirciema Str., LV-1007 Riga, Latvia; 2 AIWA Clinic, 241 Latgales Str., LV-1019 Riga, Latvia; 3 Latvian Biomedical Research and Study Centre, 1 Ratsupites Str., LV-1067 Riga, Latvia; 4 Faculty of Medicine, Riga Stradiņš University, 16 Dzirciema Str., LV-1007 Riga, Latvia; 042317@rsu.edu.lv; 5 Centrālā Laboratorija, 4a Grēdu Str. LV-1019 Riga, Latvia; 6 Department of Biology and Microbiology, Riga Stradiņš University, 16 Dzirciema Str., LV-1007 Riga, Latvia; 7 Department of Infectology, Riga Stradiņš University, 16 Dzirciema Str., LV-1007 Riga, Latvia; 8 Institute of Food Safety, Animal Health and Environment BIOR, 3 Lejupes Str., LV-1076 Riga, Latvia

**Keywords:** adenoids, otitis media with effusion, adenoid hypertrophy, microbiome, *16S rRNA* gene V3-4 sequencing

## Abstract

**Background:** The adenoid surface serves as a potential bacterial reservoir for upper respiratory tract pathologies, including Adenoid Hypertrophy (AH) and Otitis Media with Effusion (OME). While dysbiosis is implicated in the pathogenesis of these conditions, it remains unclear whether the microbiome of children with otitis media differs significantly from those with simple hypertrophy when compared against a healthy baseline. This study aimed to characterize the adenoid microbiome in children with adenoid hypertrophy with and without middle ear effusion, contrasting them with a healthy control group. **Methods:** We conducted a cross-sectional case–control study involving 58 pediatric participants divided into three groups: adenoid hypertrophy, adenoid hypertrophy and otitis media with effusion and healthy controls. Nasopharyngeal swabs were collected, and bacterial community composition was analyzed using *16S rRNA* gene V4 sequencing. Alpha and beta diversity metrics, taxonomic composition, and differential abundance were evaluated. **Results:** Alpha diversity was significantly reduced in both disease groups compared to healthy controls (*p* < 0.001), indicating a collapse of microbial richness and evenness. Beta diversity analysis revealed a distinct separation between healthy and diseased cohorts; however, the microbial profiles of the AH and AH with OME groups were nearly identical. Taxonomic analysis demonstrated that disease was characterized by the depletion of health-associated commensals, specifically *Veillonella*, *Actinomyces*, and *Gemella*, and a concomitant expansion of *Fusobacterium*, *Haemophilus*, and *Streptococcus*. **Conclusions:** Adenoid hypertrophy is associated with a profound dysbiotic shift characterized by the loss of protective commensal flora and the dominance of pathobionts. The lack of significant microbial differentiation between the AH and AH with OME groups suggests that the adenoid surface acts as a centralized reservoir of dysbiosis, the composition of which is conserved regardless of the presence of middle ear effusion. These findings highlight the importance of restoring the commensal microbiome in the management of chronic adenoid disease.

## 1. Introduction

The upper respiratory tract is constantly exposed to inhaled particles and microorganisms; consequently, its mucosa has evolved to function as both a physical barrier and an immunologically active interface [1,2]. This compartment is non-sterile, hosting diverse niche-specific microbial communities that interact physically and immunologically with the host epithelium [1,2]. These communities contribute fundamentally to mucosal homeostasis and immune education throughout childhood [1].

The composition of the upper-airway microbiota is dynamic, shaped by factors including age-dependent immune maturation, viral exposures, antibiotic use, and lifestyle [1,3]. In healthy subjects the upper airway is typically dominated by phyla such as *Firmicutes*, *Proteobacteria*, *Bacteroidota*, and *Actinobacteriota*, with recurrent genera including *Streptococcus*, *Haemophilus*, *Moraxella*, *Staphylococcus*, *Corynebacterium*, and *Dolosigranulum* [3]. Clinical complications may arise not only from the invasion of specific pathogens but also from shifts in community structure—termed dysbiosis—which can modulate inflammatory tone and susceptibility to infection [1,2].

Within this ecosystem, the pharyngeal tonsil occupies a critical anatomical niche within Waldeyer’s ring, acting as a primary site for antigen sampling at the nasopharyngeal crossroad. Their reticular epithelium and lymphoid architecture support local immune surveillance; however, the crypt organization also renders the adenoid a potential reservoir for microbial persistence and chronic inflammation [4,5]. Given their proximity to the Eustachian tube orifice, colonization of the adenoid by bacteria may impair middle-ear ventilation via both mechanical obstruction and biological pathways (e.g., mucosal oedema and inflammatory signalling), providing a plausible link between nasopharyngeal dysbiosis and middle-ear disease [4,6,7].

Otitis media with effusion (OME), defined by the presence of middle-ear fluid without acute signs of infection, is highly prevalent in childhood [6,8]. While spontaneous resolution is common, recurrent or persistent effusion in the middle ear can lead to conductive hearing loss, potentially impacting speech and language development. The pathophysiology is multifactorial, but Eustachian tube dysfunction remains a central pathway [6,7,8]. Clinical management reflects this complex relationship: while tympanostomy tubes are frequently utilized to address hearing loss, adjuvant adenoidectomy is often considered to address the underlying reservoir of inflammation, consistent with current guidelines [6,7,8].

A microbial contribution to chronicity is well-recognized; classical otopathogens (*Haemophilus influenzae*, *Streptococcus pneumoniae*, *Moraxella catarrhalis*) are frequently implicated. However, their simple detection does not explain why effusion persists in some children but resolves in others [6,9,10]. A leading hypothesis involves biofilm formation, where bacteria embedded in an extracellular matrix evade host defences and sustain low-grade inflammation [7,11,12]. Indeed, previous studies have reported higher adenoid biofilm burdens in children with OME compared to those with hypertrophy alone [11,12].

While traditional culture remains valuable for susceptibility testing, it often underestimates polymicrobial communities and fastidious organisms [2]. Culture-independent approaches, such as 16S rRNA gene amplicon sequencing, enable broad-spectrum profiling of bacterial diversity and relative abundance [2]. According to prior research, the present study applies 16S rRNA gene sequencing to characterize the adenoid surface microbiome. Uniquely, we compare bacterial communities across three distinct pediatric cohorts—Adenoid Hypertrophy, Adenoid Hypertrophy with OME, and healthy controls—to identify dysbiotic shifts and potential microbial drivers of disease persistence [9,10].

## 2. Materials and Methods

### 2.1. Ethics Statement

Our research received the official approval of Riga Stradiņš university ethics committee (approval nr. 2-PE K-4/264/2022). All participants signed an informed consent. All procedures were conducted in accordance with the Helsinki declaration on biomedical studies.

### 2.2. Patient Selection, Inclusion and Exclusion Criteria

In this study, we analyzed adenoid surface swabs obtained from patients aged 3 to 9 years. The patients were divided into two study groups: those with adenoid hypertrophy and otitis media with effusion, and those with adenoid hypertrophy without middle ear effusion. A third-control group consisting of nasopharyngeal swabs collected from healthy children aged 5 to 7 years presenting for routine pre-school health check-ups.

Samples for the two study groups were prospectively acquired from consecutive eligible patients during adenoidectomies performed under general anesthesia at a single center (Multifunctional Surgical Clinic “AIWA”) between 2022 and 2025. All surgical procedures were performed by the same surgeon using a standardized technique: adenoidectomy using a curette with visualization via a laryngeal mirror.

#### 2.2.1. Inclusion Criteria for the First Study Group—Children with Adenoid Hypertrophy but No Middle Ear Effusion

- Age: 3 to 9 years old.
- Clinical Findings: Endoscopic evidence of grade II, III, or IV adenoid hypertrophy, and/or confirmation via computed tomography (CT) or magnetic resonance imaging (MRI).
- Tympanometry: Type A tympanogram bilateral.
- Surgical Protocol: Scheduled for adenoidectomy under general anesthesia.
- Ethical Compliance: Written informed consent was obtained from the patients’ parents or legal guardians.

#### 2.2.2. Inclusion Criteria for the Second Study Group—Children with Adenoid Hypertrophy and Bilateral or Unilateral Middle Ear Effusion

- Age: 3 to 9 years old.
- Clinical Findings: Endoscopic evidence of grade II, III, or IV adenoid hypertrophy, and/or confirmation via computed tomography (CT) or magnetic resonance imaging (MRI).
- Tympanometry (OME Group): Type B tympanogram (unilateral or bilateral).
- Surgical Protocol: Scheduled for adenoidectomy under general anesthesia.
- Ethical Compliance: Written informed consent was obtained from the patients’ parents or legal guardians.

#### 2.2.3. Inclusion Criteria for the Control Group

- Age: 3 to 9 years old.
- Clinical findings: No objective evidence of adenoid hypertrophy greater than Grade II.
- Surgical History: No history of prior adenoidectomy or middle ear surgery.
- Health Status: No history of acute upper respiratory tract infection within the preceding month.
- Tympanometry: Bilateral Type A tympanograms.

#### 2.2.4. Exclusion Criteria for Study Groups

- Patients falling outside the specified age range.
- Consent: Refusal of the patient or legal guardian to participate in the study.
- International patients traveling to Latvia specifically for surgery to minimize geographic microbiome variation.
- Clinical signs of acute upper respiratory tract infection or acute external or middle ear infection at the time of surgery. Signs defined as: fever, cough, purulent nasal discharge, odynophagia (painful swallowing), pharyngeal hyperemia, or tonsillar exudate.
- Administration of systemic or topical antibiotics within the two weeks prior to sample collection.
- Patients with conditions affecting immune competence, including HIV/AIDS, Viral hepatitis (A, B, or C), Malignancies of the nasopharynx, ongoing chemotherapy, Diabetes mellitus, Chronic autoimmune diseases (e.g., Sarcoidosis, Granulomatosis with polyangiitis).
- Current or recent use of probiotics or other microbiome-altering substances.
- Congenital craniofacial malformations (e.g., cleft lip/palate).

### 2.3. Sample Collection for the Study Groups

Samples for the study groups were collected from the surface of the adenoid tissue removed during adenotomy procedures. All adenotomies were performed under general anesthesia. Following induction, the patient was intubated using a sterile endotracheal tube. To minimize contamination of the adenoid tissue by buccal or dental microflora during removal, the perioral region, lips, and oral cavity were carefully disinfected with a chlorhexidine-based solution three times.

The adenoid was resected using a curette under visual guidance by a laryngeal mirror tilted upward. The adenoid tissue was removed through the oral cavity without contacting the surrounding oral mucosa or teeth. The resected adenoid was immediately placed on a sterile field. A sterile swab was applied to the adenoid surface and rotated several times to ensure adequate sample collection. The swab was then placed into a COPAN eNat^®^ transport system (Copan, Brescia, Italy) and immediately transported to the Latvian Biomedical Research and Study Centre for preservation and subsequent sequencing.

### 2.4. Sample Processing and Sequencing

Microbial DNA from the adenoid samples was extracted using the FastDNA Spin Kit for Soil (MP Biomedicals, Santa Ana, CA, USA) according to the manufacturer’s instructions. The V3–V4 hypervariable regions of the bacterial *16S rRNA* gene were amplified using the primer pair 341F/805R and Phusion U Multiplex PCR Master Mix (Thermo Fisher Scientific, Waltham, MA, USA). PCR reactions were performed under the following conditions: 98 °C for 30 s, 35 cycles of denaturation at 98 °C for 10 s, annealing at 67 °C for 15 s and extension at 72 °C for 15 s, followed by a final extension at 72 °C for 7 min. In a second PCR step, custom-ordered unique Illumina dual-index barcodes (Metabion International AG, Planegg, Germany) were attached to each sample using the same master mix and cycling conditions. Two negative controls were included in each PCR run and assessed by agarose gel electrophoresis; one negative control per batch (three batches in total) was additionally included in sequencing. Amplicons were purified after each PCR step using magnetic bead-based purification (Macherey-Nagel, Düren, Germany). DNA and amplicon yield was quantified using a Qubit Fluorometer (Thermo Fisher Scientific, Waltham, MA, USA), and fragment size distribution were evaluated with an Agilent 2100 Bioanalyzer (Agilent, Santa Clara, CA, USA). Sequencing was carried out on an Illumina MiSeq platform (Illumina, San Diego, CA, USA) employing the MiSeq Reagent Kit v2 (500-cycles), generating at least 100,000 paired-end reads per sample.

### 2.5. Data Analysis

Raw sequence reads were quality filtered and trimmed using fastp tool v.0.24.0 [13]. All quality-approved sequences were imported into QIIME2 v.2025.7 [14] for subsequent analysis. The DADA2 plugin v.2025.7.0 [15] was employed for quality control and to merge paired-end reads; phiX and chimeric sequences were removed using the pooled consensus method. Amplicon Sequence Variants (ASVs) obtained from DADA2 were used to construct a phylogenetic tree with FastTree2 [16]. Taxonomic classification of ASVs was performed using a pretrained scikit-learn naïve Bayes classifier [17] based on the Silva v.138.1 reference database at 99% sequence identity [18]. Potential library preparation contaminants were identified using the decontam v.1.13.0 package [19] based on the frequency method, which detects features whose abundance varies inversely with DNA concentration. Following contaminant identification and removal, all negative control samples were excluded from the dataset. Low-abundance features were the filtered out using the following thresholds: a taxon had to be present in more than two samples, contain at least five reads per sample, and exceed a total of 10 reads across all samples. The resulting dataset was rarefied to 5000 reads per sample using the phyloseq v.1.53.0 package [20]. To ensure that the chosen rarefaction threshold was not overly stringent, sampling completeness was assessed using Good’s coverage to estimate the proportion of the total community represented by the observed sequences in each sample.

Taxonomic composition at the genus and phylum level were visualized using the microViz v. 0.12.7 [21] package. Shannon diversity and Pielou’s evenness indices were calculated using the microbiome v.1.31.4 package. Differences in alpha diversity between study groups were assessed using the Wilcoxon rank sum exact test, with *p*-values adjusted for multiple comparisons using the Benjamini–Hochberg method as implemented in the vegan v.2.7-1 package. For beta diversity, genus level abundance data were centered log—ratio (CLR) transformed, and Euclidean (Aitchison) distances were computed between samples. Microbial community composition was visualized using Principal Coordinates Analysis (PCoA) implemented in the microViz package v.0.12.7. Differences in community composition among groups were assessed using a permutational multivariate analysis of variance (PERMANOVA) with 999 permutations based on Euclidean distances. A permutation test for homogeneity of multivariate dispersions (PERMDISP), also based on Euclidean distances and 999 permutations, was used to evaluate whether observed group differences could be influenced by within group variability. Differential abundance analysis across all taxonomic ranks was conducted with the MicrobiotaProcess v.1.21.0 [22] package. Briefly, the Kruskal–Wallis test (α = 0.05) was applied to identify taxa with significant differences among groups, followed by pairwise Wilcoxon tests (α = 0.01) to determine subclass specific differences consistent with overall trends. Significantly different taxa were subsequently evaluated using linear discriminant analysis (LDA), with an effect size threshold of LDA > 3.

### 2.6. AI Statement

During the preparation of this manuscript, the authors used Google Gemini AI PRO to assist with text formatting and language editing. The authors have reviewed and edited the output and take full responsibility for the content of this publication.

## 3. Results

### 3.1. Population Statistics

A total of 58 samples were included in the study. The cohort was divided into three groups: the first study group comprised 20 children with adenoid hypertrophy and no middle ear effusion; the second study group consisted of 19 children with adenoid hypertrophy and otitis media with effusion (OME); and the control group included 19 samples from healthy subjects.

The study population consisted of 31 males and 27 females. As the age distribution was non-normal (Shapiro–Wilk test *p* < 0.001), descriptive statistics are reported as medians and interquartile ranges (IQR). The median age of the cohort was 4.0 years (IQR: 3.0). Detailed demographic characteristics are presented in Table 1.

Sequencing analysis was successful for all samples, yielding an average of 206,203.9 ± 196,343.5 sequences per sample (min: 23,405; max: 734,253). After quality control, including removal of chimeric and contaminant sequence, an average of 84,387.6 ± 87,504.2 sequences per sample were retained (min: 5076; max: 315,774). The dataset was subsequently rarefied to 5000 reads per sample. Despite this normalization, Good’s coverage remained high, with an average of 98.7% ± 0.8% (min: 96.4%; max: 99.8%), indicating that rarefaction had a minimal impact on community representation, retaining nearly all bacterial taxa present in the samples.

### 3.2. Alpha Diversity

**Figure 1:**
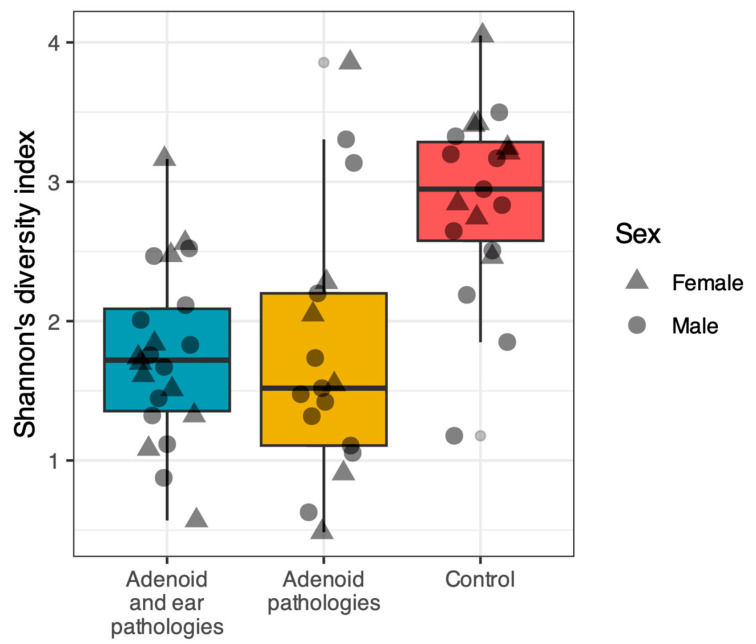
Shannon’s diversity index across study groups. Comparison of alpha diversity (richness) between patients with Adenoid Hypertrophy with OME (Blue), Adenoid Hypertrophy alone (Yellow), and control group (Red). The Control group exhibited a higher bacterial diversity compared to both study groups (*p* < 0.01). No significant difference was observed between the two study groups (*p* = 0.58). Horizontal lines within the boxplots represent the median; the lower and upper hinges correspond to the first and third quartiles (IQR). Individual data points are shaped by gender (Circle = Male, Triangle = Female).

**Figure 2:**
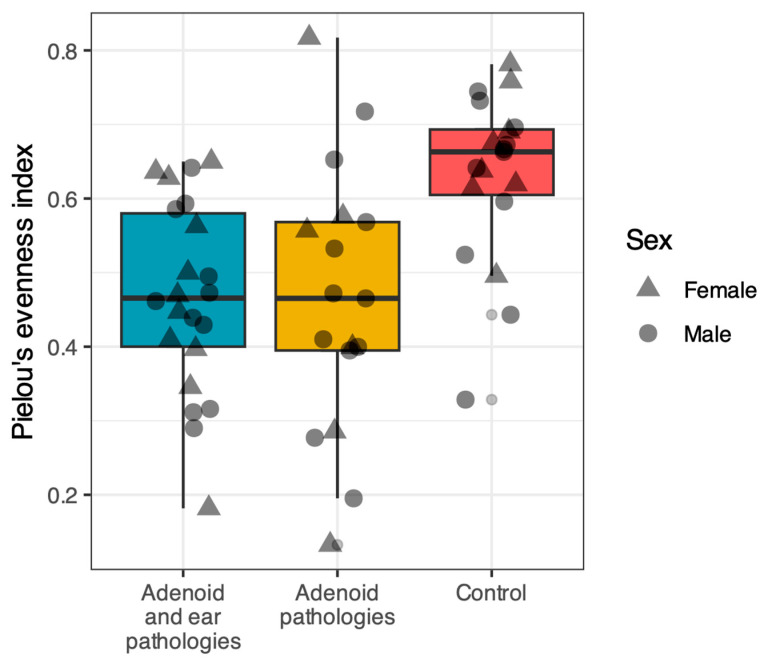
Pielou’s evenness index across study groups. Comparison of community evenness between patients with Adenoid Hypertrophy with OME (Blue), Adenoid Hypertrophy alone (Yellow), and Healthy Controls (Red). The Control group demonstrated significantly higher evenness compared to the Adenoid Hypertrophy (*p* = 0.003) and Adenoid Hypertrophy with OME (*p* < 0.01) groups, indicating a more balanced microbial community in healthy children. No significant difference was found between the two disease groups (*p* = 0.81). Horizontal lines represent the median; shapes indicate gender (Circle = Male, Triangle = Female).

Alpha diversity was evaluated using the Shannon diversity index and Pielou’s evenness index. A comparison of the three groups revealed that the nasopharyngeal microbiome of healthy controls was significantly more diverse and even compared to patients with adenoid pathology (Figure 1 and Figure 2).

Pairwise comparisons using the Wilcoxon rank-sum test (adjusted with the Benjamini–Hochberg method) confirmed that the Control group had significantly higher Shannon diversity compared to both the Adenoid Hypertrophy group (*p* < 0.001) and the Adenoid Hypertrophy with OME group (*p* < 0.001). Similarly, Pielou’s evenness was significantly higher in Controls compared to the Adenoid Hypertrophy (*p* = 0.003) and Adenoid Hypertrophy with OME (*p* < 0.001) groups.

No statistically significant differences were observed between the two disease groups (Adenoid Hypertrophy vs. Adenoid Hypertrophy with OME) for either the Shannon index (*p* = 0.58) or Pielou’s evenness (*p* = 0.81).

### 3.3. Beta Diversity

Beta diversity analysis was performed to evaluate differences in microbial community structure among the study groups using Euclidean (Aitchison) distances calculated from CLR-transformed genus-level data. Permutational multivariate analysis of variance (PERMANOVA) revealed that the microbial community structure of the Control group differed significantly from that of the Adenoid Hypertrophy group (*p* = 0.001) and the Adenoid Hypertrophy with otitis media group (*p* = 0.001). In comparison, no statistically significant difference was observed between the two pathology groups (*p* = 0.86). To assess whether these differences could be influenced by variation in within-group heterogeneity, a permutation test for homogeneity of multivariate dispersions (PERMDISP) was performed. This analysis revealed significant differences in beta diversity dispersion among groups (*p* = 0.002), with Control samples exhibiting significantly different within-group variability compared to both the Adenoid Hypertrophy group (*p* = 0.002) and the Adenoid Hypertrophy with otitis media group (*p* = 0.001). No significant difference in dispersion was observed between the two Adenoid groups (*p* = 0.533).

**Figure 3:**
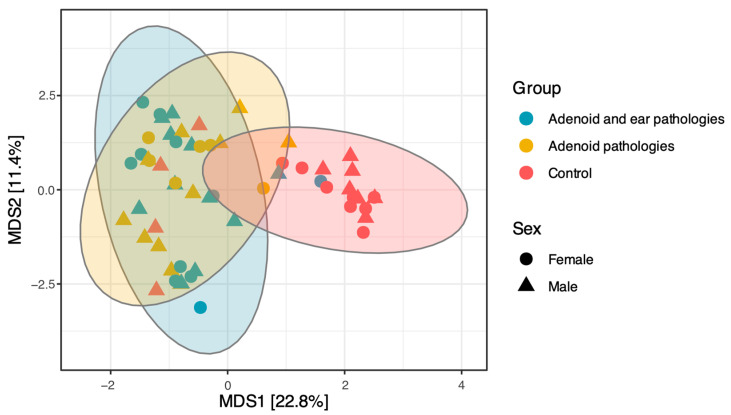
Principal Coordinates Analysis (PCoA) of the nasopharyngeal microbiome. PCoA based on Euclidean distances calculated from CLR-transformed genus-level abundance data showing differences in microbial community composition among the three study groups: Adenoid hypertrophy and middle ear effusion (Blue), Adenoid hypertrophy alone (Yellow), and Healthy Controls (Red). The Control group separates distinctly from the disease groups along MDS1, which explains 22.8% of the total distance, while the two disease groups show significant overlap, indicating a similar community structure. Shapes represent gender (Triangle = Male, Circle = Female). Ellipses represent the 95% confidence interval for each group.

These findings, consistent with the clustering patterns observed in the Principal Coordinates Analysis (PCoA; Figure 3), suggest that adenoid pathology is associated with a distinct shift in microbiome structure compared to healthy controls, whereas the presence of middle ear effusion does not confer an additional distinguishing impact on beta diversity.

PCoA revealed distinct clustering patterns corresponding to health status (Figure 3). The first principal coordinate (MDS1), explaining 22.8% of the total distance, clearly separated the Control group (Red) from the two pathology groups.

As shown in Figure 3, the Control group clustered distinctly along the positive axis of MDS1, indicating a unique microbial community structure associated with healthy children. In contrast, the Adenoid Hypertrophy and Adenoid Hypertrophy with OME groups clustered together along the negative axis of MDS1, exhibiting extensive overlap. This significant overlap suggests that the nasopharyngeal microbiome structure in children with adenoid hypertrophy is relatively conserved regardless of the presence or absence of effusion in the middle ear.

### 3.4. Taxonomy

To identify specific bacterial taxa we performed Differential Abundance Analysis using Linear Discriminant Analysis (LDA) Effect Size (LEfSe) with a threshold of LDA > 3.0.

**Figure 4:**
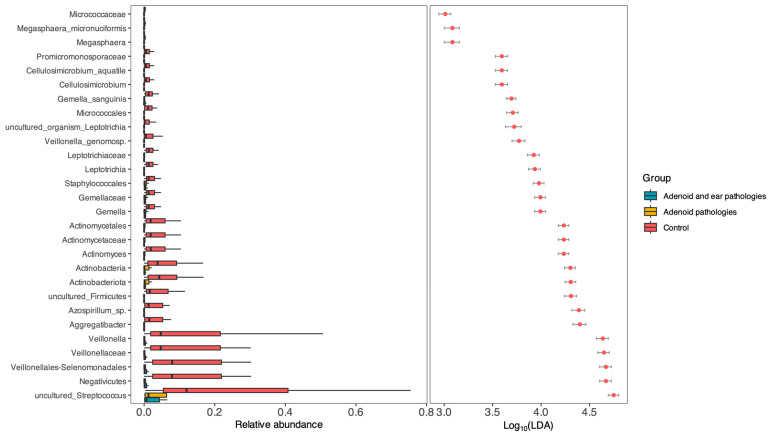
Differential abundance of bacterial taxa identified by LEfSe. (**Left**) Relative abundance boxplots and (**Right**) Linear Discriminant Analysis (LDA) scores of the most differentially abundant taxa (LDA score > 3.0). Red bars/dots represent taxa enriched in the Control group; no taxa met the threshold for enrichment in the study/disease groups. The results indicate a significant depletion of commensal genera such as Veillonella, Actinomyces, and Gemella in patients with adenoid pathology with or without middle ear effusion compared to healthy controls.

As shown in Figure 4, the analysis revealed that the vast majority of discriminative taxa were significantly enriched in the Control group (Red). Genera typically associated with a healthy commensal nasopharyngeal flora—*Veillonella*, *Actinomyces*, *Gemella*, and *Streptococcus* species—were found in high abundance in healthy subjects but were significantly depleted in both the Adenoid Hypertrophy and Adenoid Hypertrophy with OME groups.

Notably, no single taxon was found to be significantly enriched in the disease groups (Adenoid Only or Adenoid + OME) at this effect size threshold.

**Figure 5:**
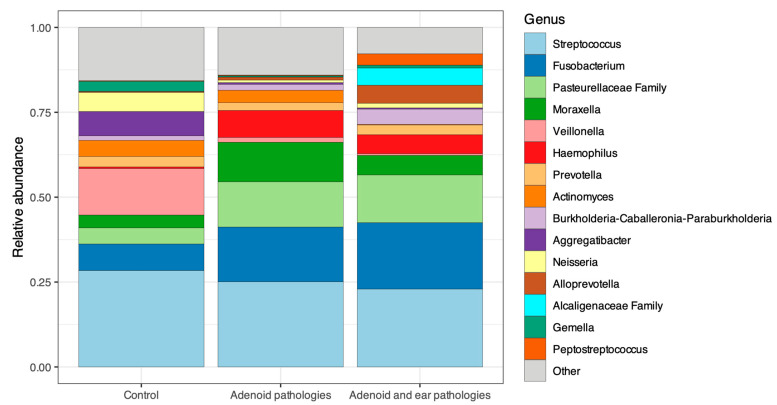
Taxonomic composition of the nasopharyngeal microbiome at the genus level. Stacked bar chart showing the average relative abundance of the top 15 bacterial genera across all three groups: Control, Adenoid hypertrophy, and Adenoid hypertrophy with middle ear effusion. “Other” represents genera with lower relative abundance.

The taxonomic composition of the microbiota of the adenoid’s surface and the nasopharynx (in controls) at the genus level is visualized in Figure 5. The relative abundance profiles reveal a marked shift in community structure between healthy controls and patients with adenoid pathology with and without middle ear effusion.

The Control group exhibited a more balanced distribution of bacterial genera, with a higher relative abundance of commensal taxa such as *Veillonella*, *Actinomyces*, *Neisseria*, and *Prevotella*. In contrast, the two disease/study groups were characterized by a reduction in these commensal genera and concomitant bacteria. Specifically, the disease groups showed increased relative abundances of *Fusobacterium* and *Haemophilus*, alongside a persistent dominance of *Streptococcus*.

**Figure 6:**
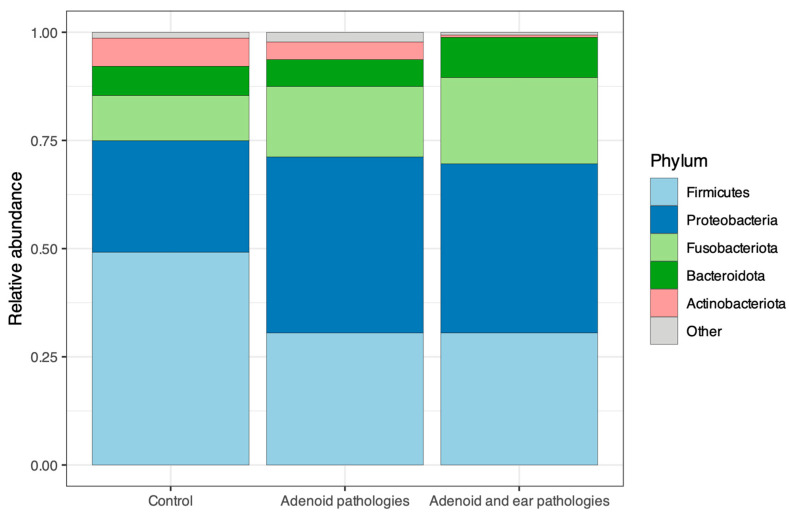
Taxonomic composition of the nasopharyngeal microbiome at the phylum level. Stacked bar chart showing the relative abundance of the top 5 most prevalent bacterial phyla across the three study groups: Control, Adenoid hypertrophy, and Adenoid hypertrophy with middle ear effusion.

The microbial community structure at the phylum level revealed distinct patterns of colonization associated with health and disease (Figure 6). Adenoid surface microbiome and the nasopharynx was dominated by 5 distinct phyla: *Firmicutes*, *Proteobacteria*, *Fusobacteriota*, *Bacteriodota* and *Actinobacteriota*. However, the relative proportions of these phyla varied by group.

In the Control group, *Firmicutes* was the most abundant phylum, comprising approximately half of the total relative abundance, followed by *Proteobacteria* and a relatively high proportion of *Actinobacteriota* phylum compared to disease groups.

Adenoid Hypertrophy and Adenoid Hypertrophy with OME groups exhibited a marked expansion of *Fusobacteriota*, which increased from a minor constituent in healthy controls to a dominant taxon in patients. Concurrently, the relative abundance of *Actinobacteriota* and *Firmicutes* decreased in the disease groups. *Proteobacteria* remained a major constituent phylum across all cohorts but appeared in slightly elevated concentrations in the pathology groups. Consistent with beta diversity findings, the phylum-level profiles of the two disease groups were almost indistinguishable.

## 4. Discussion

Our study is focused on characterizing the adenoid surface microbiome in children with adenoid hypertrophy (with and without otitis media with effusion) and comparing it to the microbiome of the nasopharyngeal microflora of healthy children, a baseline often lacking in previous clinical cohorts [3,23]. Our primary findings reveal that adenoid hypertrophy is associated with noticeable shifts in microbial community structure, consistent with observations of dysbiosis in chronic adenotonsillar disease [24,25]. We observed a distinct change in beta diversity and a significant depletion of commensals in children with adenoid hypertrophy. Notably, this dysbiotic profile was nearly identical between patients with adenoid hypertrophy alone and those with concurrent otitis media with effusion. This suggests that the adenoid microbiome alterations are primarily driven by the hypertrophic state itself rather than the presence or absence of middle ear effusion, supporting the ‘adenoid reservoir’ hypothesis described in recent literature [26].

A key hallmark of the enlarged adenoid in our cohort was the significant loss of microbial richness and evenness. Nasopharyngeal microbiome in healthy children hosted a diverse ecosystem, whereas the microbiome of the enlarged adenoid harbored a community dominated by a fewer taxa. This aligns with previous studies suggesting that high microbial diversity is a marker of upper respiratory tract health [3,23]. The reduction in Shannon and Pielou indices in our study mirrors findings by Johnston et al. [24] and Ren et al. [27], who similarly reported that chronic inflammation is linked to a decrease in nasopharyngeal bacterial complexity. This loss of diversity likely compromises the natural “healthy” microbiome of the adenoid surface, allowing opportunistic pathogens to overgrow and displace the commensal flora.

Our differential abundance analysis provided a comprehensive overview of this replacement. The microbial communities on our control samples were defined by the abundance of commensal microflora, specifically *Veillonella*, *Actinomyces*, and *Gemella*. These genera are well-documented residents of the healthy oral and nasopharyngeal cavities [28]. Veillonella is hypothesized to play a critical metabolic role by metabolizing lactate produced by *Streptococci* into beneficial short-chain fatty acids, potentially stabilizing the local pH and preventing the outgrowth of acid-tolerant pathogens [29,30,31]. The depletion of these “metabolic sinks” in both of our study groups suggests that the loss of functional stability may precede pathogen overgrowth.

In contrast, the study groups were characterized by the expansion of *Fusobacterium* and *Haemophilus* genera. *Fusobacterium* is a potent biofilm producer often associated with recurrent tonsillitis and chronic adenoiditis [32]. Its co-occurrence with *Haemophilus* supports the “co-pathogen” hypothesis, where interspecies interactions in biofilms enhance bacterial persistence and resistance to host clearance.

The depletion of *Veillonella* in the disease groups suggests a disfunction in various metabolic cross-feeding networks. *Veillonella* species are lactate-fermenting anaerobes that rely on lactate produced by other bacteria, particularly *Streptococci* [33]. In a healthy ecosystem, *Veillonella* acts as a ‘metabolic sink,’ removing the accumulation of lactic acid and thereby stabilizing the local pH. The loss of this pH-buffering capacity in the disease groups—despite the continued dominance of lactate-producing *Streptococci*—may lead to a localized acidification on the surface of the adenoid. We postulate that this acidic environment may contribute to epithelial damage, impair ciliary function, and favor the overgrowth of acid-tolerant pathobionts, thereby perpetuating the cycle of inflammation and hypertrophy [34,35].

One of the most clinically relevant findings of our study is the similarity between the microbiomes of the adenoid surfaces in children with middle ear effusion and children with healthy middle ears. Our PCA and PERMDISP analyses indicated an almost complete overlap of the bacterial communities. This challenges the assumption that children with otitis media with effusion harbor a unique or more “aggressive” adenoid microbiome than those with simple hypertrophy and no middle ear effusion [12]. Our data supports the “Adenoid Reservoir” hypothesis: the adenoid surface acts as a centralized pool of dysbiotic bacteria for all hypertrophic patients [25]. Whether this dysbiosis extends into the middle ear to cause OME may depend on mechanical factors (Eustachian tube dysfunction) or host immune factors rather than a specific shift in the adenoid bacterial community itself causing said tube dysfunction.

The findings of the current study build upon and refine our previous work in the adenoid microbiome field. In our initial pilot analysis we compared children with OME to those without middle ear effusion and observed no statistically significant differences in bacterial composition, likely due to the limited sample size (N = 19) and the absence of a healthy control group [9]. In a subsequent expanded cohort study we identified that children with OME exhibited reduced alpha diversity and an increased abundance of *Fusobacterium* compared to those with hypertrophy alone (N = 40) [10].

Present study refines these earlier findings by incorporating a control group of healthy children. By placing the disease groups in the comparison to healthy samples, we can now conclude that while subtle differences may exist between OME and non-OME clinic groups, they are overshadowed by the fundamental differences found when a healthy control group is introduced.

The impact of patient age must also be considered in the pathogenesis of OME and adenoid hypertrophy. Our study encompasses the 3–9 year age range, which covers critical developmental transitions. For instance, age significantly modifies the relationship between OME and allergic rhinitis, with a notable transition in inflammatory etiology occurring around 6 years of age [36]. Furthermore, age 4 represents a key clinical threshold where adenoidectomy plays an increasingly prominent role in OME management. While our sample size limited our ability to perform robust age-stratified subgroup analyses, it is highly likely that age-dependent immune maturation and allergic transitions exert a modifying effect on the local nasopharyngeal microbiome, warranting larger, age-stratified longitudinal cohorts.

Our study has several limitations that should be considered when interpreting the results. First, while our total cohort included 58 samples, the stratification into three subgroups resulted in relatively small sample sizes for pairwise comparisons. However, this sample size is consistent with, and in some cases exceeds, other recent *16S rRNA* gene sequencing studies of the pediatric upper respiratory tract. For example, Johnston et al. analyzed adenoid samples from a similarly sized cohort to investigate otitis media, and Ren et al. established core adenoid microbiome characteristics using a smaller discovery cohort [24,25]. The prohibitive cost and bioinformatic complexity of high throughput *16S rRNA* gene sequencing often limit the feasibility of large-scale clinical studies. Despite the modest sample size, the effect sizes observed in our differential abundance analysis (LDA scores > 3.0) were robust, suggesting that the biological signal of dysbiosis in adenoid hypertrophy is strong enough to be detected even in smaller cohorts.

Furthermore, literature suggests that biofilm-producing bacteria exhibit topographic variation across different subsites of the adenoid [37]. Our reliance on surface swabbing may capture a different microbial profile than deep crypt biopsies, potentially underestimating the total polymicrobial burden residing within the tissue.

We acknowledge that sampling the adenoid surface in the disease groups versus the nasopharyngeal wall in the control group introduces a potential anatomical confounder. However, obtaining direct adenoid surface swabs from healthy, asymptomatic children is ethically dubious as it requires general anesthesia. Additionally, while the disease group spans a wider clinical age range (3–9 years) than the healthy controls (5–7 years), both represent the early school-age demographic where the microbiome is relatively stabilized, though age-dependent maturation cannot be entirely ruled out as a minor influencing factor.

Our second limitation is based on a cross-sectional design, which allows us to identify associations but prevents us from establishing causality. It remains unclear whether the observed dysbiosis (depletion of commensals and expansion of *Fusobacterium*) is the primary driver of adenoid hypertrophy or a secondary consequence of the hypoxic microenvironment. Longitudinal studies tracking the nasopharyngeal microbiome from infancy through the development of adenoid pathology would be required to definitively answer this chicken-and-egg question.

In our study we relied solely on *16S rRNA* gene V3-4 amplicon sequencing, which provides excellent genus-level identification but lacks the discriminatory power to identify specific bacterial strains. This is particularly relevant for the *Streptococcus* genus, which includes both commensals (e.g., *S*. *mitis*) and potential pathogens (e.g., *S*. *pneumoniae*, *S*. *pyogenes*). Future studies utilizing “shotgun” metagenomics could provide the strain-level granularity needed to further dissect the pathogenic potential of the “streptococcal expansion” observed in our disease groups, however “shotgun” studies are at least twice as expensive per sample.

Finally, while this study focused on adenoid hypertrophy and OME, future research should expand to encompass pediatric chronic rhinosinusitis. The adenoid reservoir likely plays a parallel role in pediatric chronic rhinosinusitis, and investigating these overlapping pathologies in the context of professional consensus guidelines could provide a more comprehensive understanding of pediatric upper airway dysbiosis [38].

## 5. Conclusions

Adenoid hypertrophy is associated with a shift in microbiological composition on the surface of the adenoid. Our results indicate a collapse of microbial diversity and a significant depletion of commensals, such as *Veillonella* and *Actinomyces*, rather than solely the proliferation of specific pathogens.

A lack of significant differences in microbial community structure on the surface of the adenoid between children with adenoid hypertrophy alone and those with concurrent otitis media with effusion suggests that the adenoid microbiome remains relatively stable regardless of middle ear status.

These conclusions should be interpreted in the context of the study’s cross-sectional design and the sample size limitations inherent to pediatric surgical cohorts.

## Figures and Tables

**Table 1:** Descriptive statistics.

						Shapiro–Wilk	
	N	Median	SD	IQR	Range	W	*p*
Age	58	4.00	1.80	3.00	6	0.864	<0.001

## Data Availability

All data are available upon reasonable request from the corresponding author.
